# Online inference making and comprehension monitoring in children during reading: Evidence from eye movements

**DOI:** 10.1177/1747021821999007

**Published:** 2021-03-15

**Authors:** Holly Joseph, Elizabeth Wonnacott, Kate Nation

**Affiliations:** 1 Institute of Education, University of Reading, Reading, UK; 2 Department of Education, University of Oxford, Oxford, UK; 3 Department of Experimental Psychology, University of Oxford, Oxford, UK

**Keywords:** Reading, eye movements, inference making, comprehension monitoring, children, open science

## Abstract

Inference generation and comprehension monitoring are essential elements of successful reading comprehension. While both improve with age and reading development, little is known about when and how children make inferences and monitor their comprehension during the reading process itself. Over two experiments, we monitored the eye movements of two groups of children (age 8–13 years) as they read short passages and answered questions that tapped local (Experiment 1) and global (Experiment 2) inferences. To tap comprehension monitoring, the passages contained target words which were consistent or inconsistent with the context. Comprehension question location was also manipulated with the question appearing before or after the passage. Children made local inferences during reading, but the evidence was less clear for global inferences. Children were sensitive to inconsistencies that relied on the generation of an inference, consistent with successful comprehension monitoring, although this was seen only very late in the eye movement record. Although question location had a large effect on reading times, it had no effect on global comprehension in one experiment and reading the question first had a detrimental effect in the other. We conclude that children appear to prioritise efficiency over completeness when reading, generating inferences spontaneously only when they are necessary for establishing a coherent representation of the text.

## Introduction

Reading comprehension is complex and multifaceted (Castles et al., 2018). It is dynamic, meaning that as people read, they need to construct and revise a mental representation of the text, often referred to as a situation model (Graesser & Clark, 1985; Graesser et al., 1994; Kintsch, 1998). Key to building a coherent and well-specified situation model is the ability to draw inferences and to monitor comprehension. Although these processes have been studied extensively in the literature on skilled processing, less is known about children’s processing. This is surprising, given the suggestion that inference generation and comprehension monitoring might be difficult for younger children, and be a particular locus of difficulty for some children who find reading comprehension difficult (Cain & Oakhill, 1999; Oakhill et al., 2005; Yuill & Oakhill, 1988). In this study, we used eye movement methodology to investigate these processes in real time, as children read texts that required inferences to be made.

### Inference making

Inference generation is critical for successful text comprehension. Skilled readers routinely draw on multiple sources of information as they read; integrating, suppressing, and elaborating incrementally and dynamically to maintain a coherent situation model. Inferences allow readers to use information that is in the text, and to enrich it by connecting it with background knowledge to go beyond what is explicitly stated (Graesser et al., 1994).

A key distinction is between local and global inferences. Local inferences tend to require the integration of two propositions within a text and are typically cued by a pronoun, synonym, or category exemplar, for example, *The children took the dog for a walk. Suddenly it* (pronoun)/ *the hound* (synonym), *the animal* (category exemplar) *ran off*. Here, a local inference makes a mapping between the antecedent (*dog*) and its anaphor (*it*, *hound*, or *animal*). This type of inference involves information that is still active in working memory (Albrecht & O’Brien, 1993), and it has been argued that it requires relatively little processing effort (Graesser et al., 1994). Consistent with this, empirical data show that local inferences are indeed processed online (i.e., in real time, as the reader reads relevant parts of text that might trigger the inference) by skilled adult readers (Magliano et al., 1993; McKoon & Ratcliff, 1992). One example of this comes from Duffy and Rayner’s (1990) eye movement study in which adults read sentences containing a typical (*doctor*) or atypical (*writer*) antecedent of a target anaphor (*profession*). Reading times on the anaphor were longer following atypical than typical antecedents indicating that this type of inference is made spontaneously as people read and that atypical antecedents are more difficult to link to their anaphor.

In contrast to local inferences, global inferences draw on information that is not currently available to working memory but is relevant to the text being read (McKoon & Ratcliff, 1992). This might be information elsewhere in the text or information retrieved from background knowledge. One important question about global inferences is whether they are made spontaneously and with minimal effort, as people read. Proponents of the minimalist theory of inference generation (e.g., Casteel & Simpson, 1991; McKoon & Ratcliff, 1992) argue that global inferences are only made spontaneously if there is a local coherence break or if global information is readily available. There is a good reason for this proposal: if all possible inferences licenced by the text were to be made, comprehension would be extremely laborious and inefficient, not to mention confusing. However, constructivist theorists argue that global inferences are made online more often than minimalists claim (e.g., O’Brien & Albrecht, 1992; van den Broek, 1990), and a number of studies have indeed shown this to be the case (Albrecht & O’Brien, 1993; Calvo & Castillo, 1996; Klin et al., 1999; Lea, 1995).

Although beyond our scope to consider all relevant studies in detail, two are particularly pertinent. To address the issue of when adults make global inferences, Calvo et al. (2001) monitored eye movements as adults read short passages such as (a) and (b) (translated here from Spanish) in which an identical target sentence was preceded by a predictive context (a) or neutral control context (b). In the predictive context condition only, second pass reading times (duration of all fixations on the target region following initial reading of the region) were longer on the non-predictable word (*slept*), and participants made more regressions out of the following region (*for an hour*). Calvo et al. argued that this pattern of effects indicated that global inferences are generated spontaneously in predictable contexts, but not immediately on reading the inducing context. Instead, they arise later in processing, as indexed by second pass reading times and reading behaviour after the target word.

(a) Predictive context: *Three days before the examination, the pupil went to the library, looked for a separate table and opened his notebook. The pupil* ***studied****/* ***slept*** *for an hour approximately.*
(b) Control context: *The pupil who was a little tired after finishing his examination forgot his notebook and left it on a table in the library. The pupil* ***studied****/* ***slept*** *for an hour approximately*.

O’Brien et al. (1988) also investigated the circumstances under which adults spontaneously generate inferences as they read. Similar to Calvo et al. (2001), they manipulated the degree to which a preceding context supported the targeted inferences. When the preceding context was predictive, in example (c), *stabbed* rather *assaulted* for the target word *knife*, participants were no slower to read the second instance of *knife* when it followed *weapon*, rather than repeated presentation of the target word itself, *knife*. This suggests that they had inferred that the weapon was indeed a knife. Two further observations are noteworthy. First, this effect was only seen when the preceding context was strongly predictive. In example (d), reading times were longer on the target word (the second instance of *diamond*) when it followed *large stone* rather than *diamond* in the first sentence. Second, even when context was supportive, the effects were not immediate, showing up only in later measures in the eye movement record.

(c) All the mugger wanted was to steal the woman’s money. But when she screamed, he *stabbed*/*assaulted* her with his *knife*/*weapon* in an attempt to quiet her. He looked to see if anyone had seen him. He threw the *knife* into the bushes and ran away.
(d) Joan was delighted when Jim gave her a ring with a *diamond*/ *large stone* in it. He had asked her to marry him, and now they were officially engaged. She went to show her father. He asked what kind of gem it was. She excitedly told him that it was a *diamond* from her boyfriend.

Turning to children, there is a reasonable sized literature examining children’s inference making using offline methods. Typically, children are asked to read short passages followed by questions that tap inferences that could be made. How well children answer the questions provides an estimate of whether they are able to generate the appropriate inference. Using this method, it has been shown that primary-aged children can make both global and local inferences (Ackerman, 1986; Cain et al., 2001; Casteel & Simpson, 1991; Oakhill, 1982, 1984; Oakhill & Yuill, 1986; Yuill & Oakhill, 1988), and that local or cohesive text-based inferences tend to be easier than global, elaborative, or knowledge-based ones (Barnes et al., 1996; Bowyer-Crane & Snowling, 2005, 2010; Carlson et al., 2014; Casteel & Simpson, 1991; McMaster et al., 2012). We also know that inference-making ability improves with age (Casteel & Simpson, 1991; Long et al., 1997; Oakhill, 1982, 1984; Paris & Lindauer, 1976; Pike et al., 2010).

Much less is known about the extent to which children make inferences online, as they read. Without such data, it is impossible to know how and when inferences are made. Are they made during the course of reading itself, or are they generated in response to being asked a question? The time course of eye movement data has the potential to be informative in this respect. We can ask whether certain regions of text receive longer reading times (associated with increased processing difficulty) and from this infer whether an inference has been made. The pattern of reading times provides additional information. For example, first pass reading times (often known as gaze durations) are thought to reflect early stages of processing such as lexical retrieval, while later measures such as go past times and total reading times are thought to reflect later processes such as integration and resolution of initial misanalysis (Rayner et al., 2006). Thus, looking closely at different components of the eye movement record at different points in the text can reveal much about inference making.

Recognising the utility of such methods, two studies have examined online processing of local inferences in children’s reading. van der Schoot et al. (2008) found that children aged 10 to 12 years showed longer first pass reading times on anaphors when they were hyponymic (snake-reptile) than when they were reiterations (snake-snake). This suggests that children made an inference in the hyponymic condition (that snakes are reptiles) and that this required additional processing time as compared with the reiteration condition. Potentially however, the difference between conditions could be attributed to a repeated name benefit (snake-snake) rather than inference making itself.

Joseph et al. (2015) found that 10- to 11-year-olds made more regressions (leftward eye movements to re-read previous portions of the text) out of an anaphor when its antecedent was an atypical category exemplar; in example (e), the word *crane* is less typical than *truck* for the category *vehicle*. Although this effect was seen in texts like (e) in which the anaphor was near to the antecedent, it was not seen for texts like (f) where the relationship was more distant. Joseph et al. concluded that children do make local inferences when they are easy to make (i.e., close in space) but not when they are more demanding, as in the distant condition. Like van der Schoot at al., inference making was seen in later eye movement measures, suggesting that inference generation is not immediate and occurs downstream of the point at which an inference could first be made.

(e) *It had been a long day. The builders were exhausted. Eventually a truck/crane arrived to help. They needed the vehicle because the load was so heavy. At last they could start work on the building*.
(f) *It had been a long day. Eventually a truck/crane arrived to help. The builders were exhausted. They needed the vehicle because the load was so heavy. At last they could start work on the building*.

Overall, there is some evidence that children make local inferences spontaneously as they read, but there is little research on global inferences, and certainly previous research does not reveal how children process local versus global inferences online and whether the text structure can help them to do so spontaneously.

### Comprehension monitoring

Comprehension monitoring refers to the processes needed to maintain relevant information, suppress no longer relevant information, and update the situation model as a person reads (Gernsbacher & Faust, 1991; Palladino et al., 2001). Comprehension monitoring is thought to encompass two separable skills: evaluation and regulation (Baker, 1985; Zabrucky & Ratner, 1992). Evaluation refers to a person’s self-assessment of understanding as they read, while regulation refers to the strategies they might use to keep comprehension on track, such as re-reading.

Comprehension monitoring in children has been measured in various ways, including conventional comprehension questions (Yuill et al., 1989), asking questions requiring (or not) a child to look back at a previous page (Garner & Kraus, 1982), analysing self-corrections (Paris & Myers, 1981), and asking the children to detect inconsistent words (Paris & Myers, 1981) or pairs of sentences that are inconsistent or contradictory (Oakhill et al., 2005). Overall, poorer readers notice fewer inconsistencies than their peers (Garner & Kraus, 1982; Oakhill et al., 2005; Paris & Myers, 1981), and are less likely to deploy strategies to resolve comprehension failure. This indicates that poor reading comprehension is associated with differences in both evaluation and regulation (Paris & Myers, 1981; Yuill et al., 1989). As with inferences, however, offline measures cannot capture comprehension monitoring precisely or directly.

Experiments with adults have used eye movement methodology to track comprehension monitoring online. The general approach has been for people to read a text that is inconsistent or anomalous with general knowledge (e.g., Paris & Myers, 1981) or with information that occurred earlier in the passage (Cook & Myers, 2004; Poynor & Morris, 2003), for example, a vegetarian eating a meaty burger (Albrecht & O’Brien, 1993). Adults exhibit longer reading times on words which are contextually inappropriate but quickly adapt their situation model to accept a new reality (e.g., once the reader has encountered a vegetarian eating a meaty burger, they are happy to accept non-vegetarian behaviour in a later event; Cook & Myers, 2004; Poynor & Morris, 2003), although this process is not always straightforward or easy (Kendeou et al., 2014).

A number of studies have also examined the online detection of inconsistencies during reading in children (Connor et al., 2015; Ehrlich et al., 1999; van der Schoot et al., 2009, 2012; see also Vorstius et al., 2013). Ehrlich et al. used self-paced reading to examine whether skilled and less skilled comprehenders aged 9 to 10 years detected inconsistencies in a text in which target anaphors were either repeated noun phrases or inconsistent noun phrases. Reading times on target anaphors were longer when they were inconsistent with previous text, providing evidence that children do monitor their comprehension online. However, reading time data were collected during the second reading of the text, and we therefore cannot know whether inconsistencies were detected spontaneously during initial reading. We also do not know whether the effect was due to simply comparing repeated versus non-repeated target words across the two conditions. We will return to this issue later.

Drawing on studies with adults noted above (e.g., Albrecht & O’Brien, 1993; Poynor & Morris, 2003), van der Schoot and colleagues (2012) examined comprehension monitoring in 10- to 12-year-olds as they read texts in which the action of a protagonist was consistent or inconsistent with a previous description of their character. Their findings were not robust (mostly significant in by-participant but not items analyses), but children tended to show inconsistency effects when the target sentence was near to the relevant context: that is, children (both good and poor comprehenders) spent longer reading the inconsistent than consistent sentences. In contrast, when the target sentence was further from the relevant context, only better comprehenders showed inconsistency effects. However, as target regions were entire sentences, it is not possible from this study to know exactly when during reading the inconsistencies were detected.

Finally, Connor et al. (2015) addressed the issue of the immediacy with which inconsistencies are detected. They presented 52 children (mean age = 10.6) with sentence pairs such as in example (i).

(g) *For the wedding Linda wore her best outfit. The colorful plant/dress was one of her favorites.*

The children showed longer first pass reading times on target words that were implausible (*plant*) than plausible (*dress*), suggesting that they were monitoring their comprehension online. This effect was larger for those children with strong language skills, consistent with them being more adept at comprehension monitoring. Together the studies to date suggest that children of the age of interest do evaluate their comprehension online but differ in how skilled they are at regulating it (and this relates to their own language and reading skills; Helder et al., 2016).

### Prompts and reading strategies

One question arising from the previous discussion is whether it is possible to adapt text to encourage children to make inferences and actively monitor their comprehension. As noted above, adults do not always automatically generate inferences, but asking a question during the reading episode (sometimes referred to as an embedded question) can help them to generate inferences as they read (Callender & McDaniel, 2007; Smith et al., 2010; van den Broek et al., 2001). Interestingly, although there is evidence that asking children questions after reading helps them make global inferences from what they have already read (e.g., Carlson et al., 2014; McMaster et al., 2012; Paris & Lindauer, 1976), presenting a question during reading has been shown to have a detrimental effect on younger children’s comprehension. van den Broek et al. (2001) gave children (aged 10, 13, and 16 years) and adults passages with questions presented during reading (embedded in the passages) or after reading. They found that asking questions during reading interfered with the youngest children’s recall of text information and they answered fewer questions correctly in this condition. For adolescents and adults, however, asking questions during reading resulted in higher performance in response to the comprehension questions.

Kaakinen et al. (2015) investigated whether children and adults read differently when passages were preceded by either a title (*Forests are important*) or a “why” question (*Why are forests important?*). They reasoned that a “why” question would change reading behaviour as it would encourage participants to strive for greater standards of coherence (van den Broek et al., 2011). The presence of a question resulted in faster reading of the passage for adults, and for older children (10 years upwards) whereas the younger children (8-year-olds) slowed their reading in the question condition. However, response accuracy to comprehension questions was equivalent across age group and condition: while reading a question rather than a title affected reading behaviour, it did not affect endpoint comprehension, suggesting that each age group adjusted their reading behaviour in a way that benefitted them. Indeed, the observation that 8-year-olds did not differ from adults in global comprehension suggests that slowing down in response to the question was a good thing.

In summary, although inference making and comprehension monitoring are core to reading comprehension, relatively few studies have investigated these processes directly as children read. Our first goal was to add to this evidence base. A few eye movement studies have explored children’s generation of local inferences but investigations of more global inferences are lacking. To remedy this, we targeted local inferences in Experiment 1 and global inferences in Experiment 2. Both experiments included a consistency manipulation. This allowed us to track children’s sensitivity to inconsistency and from this provide information about comprehension monitoring, in terms of both evaluation and regulation. The time course of inference and inconsistency effects is of particular interest given Connor et al. (2015) found effects of inconsistency occurred earlier in the eye movement record than the effects of inference reported in other studies with children of a comparable age (Joseph et al., 2013; van der Schoot et al., 2008). Finally, we also examined whether children adapted their reading behaviour in response to being asked a question at the start of each passage, rather than at the end. If children are reading purposefully, prior knowledge of the question should affect reading, specifically with respect to inference generation and comprehension monitoring.

### Methodological issues

Both experiments used eye movement methodology to assess comprehension processes as they happen. They draw on established psycholinguistic methods and a large evidence base which provides a good understanding of how different components of the eye movement record relate to the linguistic and cognitive processes that serve reading comprehension (Clifton et al., 2016; Rayner, 1998). As has been argued before (Joseph et al., 2013, 2015), this approach holds promise for understanding how reading comprehension develops, and how it might go astray for children who find reading comprehension difficult. This reasoning guided the development of the experiments reported in this article. Our intention was to test whether individual differences in reading comprehension (as assessed by a standardised test) are associated with differences in inference generation and comprehension monitoring (as revealed by data from the eye movement record). However, due primarily to recruitment difficulties, especially in secondary schools, we did not collect the dataset that we planned, with a large age range yet relatively few participants in the older year groups. This meant that conventional age groupings were not possible, and thus chronological age would not be an appropriate means of categorising the sample. On reflection, it would have been more realistic to restrict our recruitment to a smaller age range, concentrating on the primary school years—although we know that it is adolescent readers who are underrepresented in the literature and so we were keen to include them too. As we were unable to enter age as a categorical variable into our models, we instead entered word reading efficiency which we judged to be a better index of current reading accuracy and skill, independent of comprehension skills. To preface our findings, however, our initial approach turned out to be problematic and limited in several important ways. Some of these problems relate to broader discussions about reproducibility and open science and in this spirit, we discuss upfront issues that arose, and how we dealt with them.

A critical issue that extends well beyond our own experiments is the analytic flexibility afforded by eye movement experiments, as discussed by von der Malsburg and Angele (2017). Researchers can choose from a range of metrics (e.g., first fixation duration, gaze duration, go past duration, total reading time, number of regressions in or out). They can also choose to focus on specific areas of interest within a text, perhaps a target word, for example, and/or an antecedent or following region. This plethora of measures raises concerns about Type I error. This can be reduced by choosing particular measures/regions and designating these in advance, but without pre-registration of analysis plans, it is impossible to know whether a reported result is a consequence of outcome switching (i.e., the possibility of changing the outcomes of interest depending on the observed results, Munafò et al., 2017). If we now add in a desire to investigate developmental or individual differences, we add further degrees of researcher freedom. For example, it is perfectly possible to include age or reading ability as a continuous measure in an analysis, or to compare discrete groups; less easy though is to predict in advance what effects this will have on eye movement behaviour and without this constraint, the choice of what to analyse and what to report heightens the risk of fragile or spurious results entering the literature. It is also clear that the dangers of low statistical power have been underestimated in eye movement studies on reading in general (von der Malsburg & Angele, 2017) and this becomes more of a concern when individual differences are to be considered.

Returning to our own experiments, the available evidence base did not allow us to make specific hypotheses about how individual differences would be realised in the eye movement record. We did not pre-register our analysis plans and therefore our work must be regarded as exploratory. We found that adding age or reading level into some of the models caused problems with convergence and these were greater for some measures than others; we also saw complex variation in the pattern of results across different measures. It would be possible to “backfit” some of these observations to make theoretical sense (so-called HARKing—hypothesising after the results are known; for example, Kerr, 1998) but this would clearly be inappropriate. Another approach would be to report all results. Very quickly however, this leads to an unmanageable article, given the number of independent and dependent variables available for analysis. We decided instead to focus primarily on the experimental manipulations within each experiment, including just one covariate, word reading efficiency, in our models. In line with recommendations from von der Malsburg and Angele (2017), we applied Bonferroni corrections to reduce Type I error associated with testing multiple dependent eye-tracking measures. We do not test for individual differences in reading comprehension, as initially imagined. However, we have included a full description of the samples tested for each experiment within the article, and all data are publicly available, along with analysis scripts (https://osf.io/ngjra/). This provides a resource for secondary analysis by other researchers, and perhaps a repository that can be added to in future studies, increasing sample size and statistical power. We return to discuss some of these issues in the General Discussion when we consider the importance of pre-registration for future work.

## Experiment 1

Experiment 1 examined local inferences in which a category exemplar (e.g., *turtle*) acted as an antecedent to a subsequent semantic category (e.g., *reptile*). These are considered necessary for comprehension and adults make this type of inference (sometimes described as cohesive inferences) online (Duffy & Rayner, 1990; see also Yang et al., 2007). To test whether children make these inferences online, we compared reading times across three conditions (see Table 1): control (when the same token served as antecedent and anaphor, *turtle-turtle*), inference (when the anaphor was a category label referring back to the earlier antecedent, *turtle-reptile*), and inconsistent (when the anaphor was a category label that was inconsistent with the earlier antecedent, *turtle-mammal*). On the basis that children will also make these inferences online, we predicted longer reading times on the anaphor itself when an inference was needed (e.g., *turtle-reptile* as compared with *turtle-turtle*) although it should be noted that the control condition also included a repeated word, an issue that we will return to in the Discussion. We also predicted more regressions out of the anaphor, and more regressions into both the anaphor and antecedent to try to achieve resolution. In the inconsistent condition (turtle-mammal), we predicted a larger increase in reading times still reflecting the difficulty this posed for resolution of the anaphor. We also predicted higher accuracy in responding to comprehension questions following each passage in the control than inference condition.

**Table 1.** Example stimuli for Experiment 1. In each of the three versions of the passage, the target region is underlined and the antecedent region is in bold.

	Story	Question
*Control condition*	The children were on a school trip at the aquarium. They were watching a **turtle** swim slowly along in a large tank. The small turtle suddenly jumped out of the tank and surprised the children.	Why were the children surprised? a. the turtle jumped out of the fish tank b. they were on a school trip at the aquarium c. because they had a surprise party
*Inference condition*	The children were on a school trip at the aquarium. They were watching a **turtle** swim slowly along in a large tank. The small reptile suddenly jumped out of the tank and surprised the children.	Why were the children surprised? a. the turtle jumped out of the fish tank b. they were on a school trip at the aquarium c. they had a surprise party
*Inconsistent condition*	The children were on a school trip at the aquarium. They were watching a **turtle** swim slowly along in a large tank. The small mammal suddenly jumped out of the tank and surprised the children.	Where did the children go to on their school trip? a. aquarium b. tank c. zoo

We asked children comprehension questions about what they had read. On half the trials, the question appeared before the passage and on the other half after the passage. If children use the question to help them find relevant information (i.e., answer the question), reading times on both the antecedent and the anaphor should be shorter when the question appears before the passage, rather than after. This prediction stems from Kaakinen et al.’s (2015) study which found that the presence of a question before reading reduced reading times. As well as tracking eye movements as children read the passage, we monitored reading times on the question itself. If, inferences are made and the anaphor resolved during the course of reading, reading times should be faster for questions appearing after the passage rather than before.

Finally, we predicted an interaction between question location and inference condition such that the effect of inference would be greater (longer reading times and more regressions in the inference than control condition) when the question was presented before the text than after it. This would occur because children would make the full inference during passage reading if they had been prompted to do so by the question. In contrast, when the question was presented after the passage, we predicted that children would only make a partial inference, completing it on encountering the question afterwards.

### Method

#### Participants

Following Connor et al. (2015), we aimed to recruit more than 50 participants. Sixty-four 8- to 13-year-old children were recruited from primary and secondary schools in the south east of England. To establish that all children had sufficient word reading skill to cope with the experiment, we screened the sample using the Test of Word Reading Efficiency (TOWRE; Torgesen et al., 1999). This requires children to read aloud as many words and nonwords as possible from a list in 45 s. Two children obtained a standard score below normal range and were therefore excluded from the experiment. In addition, one child was excluded due to a data transfer error, two children were excluded due to tracker loss and 13 children were excluded because English was not their first language. This left 46 children whose data were entered into analyses (18 males). The mean age was 10.64 years (*SD* = 1.64) and all were monolingual English speakers with no known reading difficulties. All children received a sticker and a certificate to thank them for their participation.

We administered the York Assessment of Reading for Comprehension (YARC; Snowling et al., 2009) in which children are asked to read aloud two passages and then answer eight questions about each one. The questions tapped literal and inferential understanding as well as vocabulary knowledge, with approximately equal proportions of the three question types in each passage. We administered the primary school version of the YARC to children in Years 4 and 5, and the supplementary passages of the secondary version to children in Years 7 and 8. This meant that all children read the same passages to allow direct comparisons between all participants as well as administering an age-appropriate test to all children. All children scored within or above the normal range (minimum = 86, maximum = 121; see Table 2).

**Table 2.** Mean age and mean performance on standardised tests of reading and nonverbal IQ of participants in Experiment 1.

	Year 4	Year 5	Year 7	Year 8
Number (no. females)	16 (10)	13 (9)	13 (7)	4 (2)
Age	9.0 (0.29)	10.1 (0.28)	12.3 (0.21)	13.4 (0.46)
TOWRE words^a,b^	103 (6)	94 (9)	95 (10)	96 (14)
TOWRE nonwords^a,b^	101 (5)	100 (9)	101 (11)	101 (7)
YARC comprehension (primary)^a,c^	102 (11)	92 (11)	100 (9)	100 (7)
YARC comprehension (secondary)^a,c^	–	–	97 (9)	93 (10)

Standard deviations are in parentheses.

a Standard score, *M* = 100, *SD* = 15.

b TOWRE (Test of Word Reading Efficiency; Torgesen et al., 1999).

c YARC (York Assessment of Reading for Comprehension; Snowling et al., 2009).

#### Materials

Twenty-four short narrative passages were created, each between two and four sentences long (mean passage length was 191.3 characters, *SD* = 25.0; see Table 1). There were six versions of each paragraph, corresponding to the 2 (Question location: after text, before text) × 3 (Inference type: control, inference, inconsistent) within-participants design. Each child only saw one of the six versions of each passage and read an equal number of passages in each of the six conditions (i.e., four of each version). In each passage, there were three target regions: the antecedent, the anaphor, and the question itself. The antecedent (e.g., *turtle*) was identical across the three conditions. The anaphor region was controlled for word length (*p* > .6; see Table 3), but there were small differences in frequency between the control and inference conditions (*p*s < .05; Wild et al., 2012; see Table 3). It is noted though that frequency was lower in the control than the inference condition, therefore going against our predictions (of shorter reading times in the control condition). There were no other significant differences in frequency across conditions (*p*s > .05).

**Table 3.** Mean length and total frequencies of target words in the anaphor region in Experiment 1.

	Length	OCC-R^a^	OCC-W^a^
Control	7.1 (2.5)	1,158 (1,431)	7,438 (8,247)
Inconsistent	6.9 (2.0)	2,184 (2,673)	10,441 (12,784)
Inference	7.0 (1.8)	3,231 (2,904)	15,803 (16,489)

Standard deviations are in parentheses.

a Absolute token counts for target words across the Reading (OCC-R) and Writing (OCC-W) sections of the Oxford Children’s Corpus.

There was a multiple-choice question associated with each passage (see Table 1). The questions for the control and inference conditions were identical but the question for the inconsistent condition was different and related to information encountered before the inconsistency. For the control and inconsistent passages, the correct response to the question was explicitly stated in the text. However, in the inference condition, the correct response required children to make a local inference by linking the anaphor (*reptile*) with its antecedent (*turtle*).

All stimuli were pre-screened with ten 7- to 11-year-olds to ensure that passages were age-appropriate and easy to understand. For this procedure, children were asked to read one version of each passage (control, inference, or inconsistent) and to answer the question about each one. Passages and questions were retained if more than 70% of children answered the question correctly and reported no difficulty understanding. Small changes were made to the passages following this and the final set of materials is available at https://osf.io/ngjra/.

#### Apparatus

Children’s eye movements were recorded using a desktop Eyelink 1000 eye tracker (SR Research; Mississauga, Canada) as they read sentences from a computer monitor at a viewing distance of 62 cm. Each character covered 0.24° of horizontal visual angle and eye movements were monitored at a rate of 1,000 Hz to produce a sequence of fixations with start and finish times. Although children read binocularly, only the movements of the right eye were monitored.

#### Procedure

Testing took place in a quiet area close to the children’s classroom and children were seen individually. Following the TOWRE reading assessment, children sat in a customised chair in front of a computer monitor, supported by a chin rest and a forehead rest to ensure comfort and to minimise head movements. They first undertook a calibration procedure during which they looked at each of nine fixation points on the computer screen with an acceptance criterion of an average error below 0.4°. For each trial, they were instructed to look at a fixation box at the top left of the screen; contingent on their gaze to this box, the text then appeared on the screen. Children were told that they would be reading a series of paragraphs and questions displayed on the computer monitor in front of them and that they were to read the text from the top to the bottom of the screen (i.e., if the question was presented before the text, they should read the question first, and if the paragraph was presented before the question, they should read the paragraph first). The passage and the question with its three possible responses were presented simultaneously and remained visible throughout the trial. Children were asked to respond to the comprehension questions by pressing one of three buttons on a handheld gamepad controller, corresponding to answers (a), (b), and (c). The button press terminated the display. If the child did not press the button within 90 s of the text appearing, the display was automatically terminated. Although the location of the question was experimentally manipulated to appear before or after the text, the location of the three possible responses was always below the text. Paragraphs were presented in a pseudorandom order so that each child saw one of the six versions for each stimulus set but read an equal number of paragraphs of each version. Each child read two practice paragraphs followed by a total of 24 experimental paragraphs. The experimental session lasted 15 to 20 min. On a subsequent day, children completed the YARC, a standardised test of reading comprehension.

### Results

For the eye movement data, fixations longer than 1,200 ms and shorter than 80 ms were excluded from the data set. We selected a number of eye movement measures considered to reflect early and late stages of processing, based on previous studies (e.g., Joseph & Liversedge, 2013). The following eye movement measures were calculated for the antecedent and anaphor regions: gaze durations (the sum of all fixations in a region until a saccade out of the region), regressions out probability (the probability of making a leftward eye movement out of a region before leaving that region to the right), go past times (the sum of all temporally contiguous fixations including fixations after a regressive eye-movement to the left of the region, until the point of fixation progresses to the region to the right), regressions in (the probability of making a leftward eye movement into a region having already left that region to the right), and total reading times (the sum of all fixations in a region). Regressions in and total reading times were also examined in the antecedent region. Note that only gaze durations and total reading times were calculated for the question region as other measures were not meaningful given the changing location of the region. As is usually the case with eye movement data, our data were not normally distributed, so we log transformed all the reading time data which resulted in more normal distributions. For ease of interpretation, we report the untransformed means and standard deviations, but all analyses were conducted on the log transformed data.

All data were analysed in the R computing environment (R Development Core Team, 2011) using logistic/linear mixed models (Baayen et al., 2008; Jaeger, 2008; Quené & Van den Bergh, 2008). Specifically, we ran logistic mixed models with response accuracy and regressions data as the predicted variable, and linear mixed models with each of the of reading time measures of interest (gaze durations, go past time, total reading time) in each region of interest (antecedent region, anaphor region, and question region) as the predicted variable. All models included the two experimentally manipulated fixed factors (inference condition and question location) and word reading efficiency as a covariate which was centred. We included word reading efficiency as a control variable and so did not include interactions between this and our main fixed effects. We included random intercepts for participants and items and random by-participant and by-item slopes for all fixed effects (i.e., a full random slopes structure—see Barr et al., 2013) initially. We then used model comparison to decide on the most parsimonious model for the data (Bates et al., 2015; Matuschek et al., 2017). When a model did not converge, we first took out interactions between random slopes and then removed random slopes one by one (removing those that accounted for the least variance) until the model converged. R code and raw data (including age, gender, and reading comprehension alongside eye movement data) are provided as supplementary materials (https://osf.io/ngjra/). We chose not to interpret the effects of participant characteristics due to our relatively small sample.

We used sum (also known as deviation) coding for the question location variable, and successive-difference coding for inference type whereby we compared the control with the inference condition and the inference with the inconsistent condition. Regression coefficients, standard errors (*SE*), *t* (reading time measures) or *z* (regression probabilities) values, and corresponding *p* values are reported. We took a conservative approach to adjusting for multiple comparisons (following von der Malsburg & Angele, 2017) by dividing our alpha level by the number of dependent variables (usually 5) plus the number of hypotheses relating to the two independent variables (usually 3), while also accounting for a mean correlation between dependent variables of .6. We used on an online calculator (Quantitative Skills, 2020) to produce these adjusted *t* and *z* scores and they are provided under each table of model output.

#### Answers to the comprehension question

Table 4 shows the mean error rates in response to the comprehension question asked before or after each passage. Comprehension was high with a mean error rate of 16% across all conditions showing that the passages were understood well. Children made more errors when the question was presented before the text (*b* = 0.66, *SE* = 0.25, *z* = 2.63). There was no effect of inference type (*b* = 0.06, *SE* = 0.11, *z* = 0.53) and no interaction (*b* = 0.29, *SE* = 0.23, *z* = 1.26).

**Table 4.** Response error rates to comprehension questions in Experiment 1.

	Control	Inconsistent	Inference
Question after text	0.12 (0.33)	0.14 (0.35)	0.10 (0.29)
Question before text	0.22 (0.42)	0.12 (0.33)	0.24 (0.43)

#### Eye movement data

##### Antecedent region

Table 5 shows mean reading times and regression probabilities in the antecedent region, and model results are shown in Table 6. We did not include inference type in the models for gaze duration, go past times, or regressions out as paragraphs were identical at this point and so no effects were anticipated. We did, however, predict different reading patterns during first pass as a function of question location, and later effects (in total reading times and regressions in) of inference. In gaze durations, go past times, and total reading times, there was a main effect of question location with longer reading times when the question was presented after the passage. There was no main effect of inference type and no interactions. Finally, children with better word reading (as indexed by raw score on TOWRE words subtest) showed shorter go past and total reading times in the antecedent region.

**Table 5.** Mean reading times and regression probabilities for all children in the antecedent region in Experiment 1.

	Question after text			Question before text		
	Control	Inference	Inconsistent	Control	Inference	Inconsistent
Gaze durations	462 (338)	470 (408)	469 (439)	400 (287)	404 (308)	408 (336)
Go past time	780 (805)	810 (903)	801 (836)	593 (472)	773 (900)	824 (1,172)
Regressions out	0.33 (0.47)	0.33 (0.47)	0.36 (0.48)	0.28 (0.45)	0.36 (0.48)	0.34 (0.48)
Regressions in	0.22 (0.42)	0.26 (0.44)	0.25 (0.44)	0.27 (0.44)	0.21 (0.41)	0.18 (0.39)
Total reading time	683 (640)	746 (624)	732 (624)	612 (447)	647 (583)	684 (787)

Standard deviations in parentheses.

**Table 6.** Results for the antecedent region examining the effect of inference, question location, word reading skill, and comprehension skill on all eye movement measures in Experiment 1.

	Question location (before vs. after)	Inference Contrast 1 (control vs. inference)	Inference Contrast 2 (inconsistent vs. inference)
Gaze duration	*b* = .06, *SE* = .02, *t* = 2.94, *p* < .005*		
Go past time	*b* = .07, *SE* = .02, *t* = 3.30, *p* < .005*		
Regressions out	*b* = .05, *SE* = .08, *z* = 0.56, *p* > .5		
Regressions in	*b* = .09, *SE* = .08, *z* = 1.09, *p* > .2	*b* = .08, *SE* = .19, *z* = 0.44, *p* > .6	*b* = .13, *SE* = .20, *z* = 0.65, *p* > .5
Total reading time	*b* = .06, *SE* = .02, *t* = 3.40, *p* < .001*	*b* = .0004, *SE* = .04, *t* = 0.01, *p* > .9	*b* = .002, *SE* = .04, *t* = 0.04, *p* > .9
	Question × Inference Contrast 1	Question × Inference Contrast 2	Word reading efficiency
Gaze duration			*b* = .01, *SE* = .004, *t* = 2.17, *p* < .05
Go past time			*b* = .02, *SE* = .005, *t* = 4.44, *p* < .001*
Regressions out			*b* = .03, *SE* = .07, *z* = 2.22, *p* < .05
Regressions in	*b* = .27, *SE* = .19, *z* = 1.47, *p* < .1	*b* = .09, *SE* = .20, *z* = 0.46, *p* > .6	*b* = .02, *SE* = .01, *z* = 1.68, *p* = .09
Total reading time	*b* = .04, *SE* = .04, *t* = 0.87, *p* > .3	*b* = .02, *SE* = .04, *t* = 0.40, *p* > .6	*b* = .02, *SE* = .005, *t* = 3.98, *p* < .001*

*SE*: standard error.

* Two-tailed significance criterion (*t* or *z* ⩾ 2.29, *p* < .022), corresponding to a 5% error, with adjustment for multiple comparisons.

##### Anaphor region

Eye movement data for the anaphor region are shown in Table 7 and model results in Table 8. There was a reliable difference in gaze durations, go past times, regressions in and total reading times between the inference and control conditions, with longer reading times on, and more regressions into (but not out of), the inference than the control condition. However, there were no differences between the inconsistent and inference conditions in any measure, no effect of question location, and no interactions. As in the antecedent region, more efficient word readers were generally faster (in all reading time measures) and made fewer regressions out of the region.

**Table 7.** Mean reading times and regression probabilities in the anaphor region in Experiment 1.

	Question after text			Question before text		
	Control	Inference	Inconsistent	Control	Inference	Inconsistent
Gaze duration	380 (306)	442 (420)	423 (321)	310 (187)	436 (374)	445 (314)
FP regressions out	0.32 (0.47)	0.31 (0.46)	0.29 (0.45)	0.39 (0.49)	0.32 (0.47)	0.24 (0.43)
Go past time	616 (574)	768 (1,272)	703 (655)	669 (698)	724 (650)	784 (942)
Total reading time	565 (446)	739 (616)	847 (694)	537 (405)	752 (569)	707 (579)
Regressions in	0.20 (0.40)	0.25 (0.43)	0.31 (0.47)	0.15 (0.36)	0.30 (0.46)	0.22 (0.42)

*FP:* First pass. Standard deviations in parentheses.

**Table 8.** Results for the anaphor region examining the effect of inference, question location, word reading skill, and comprehension skill on all eye movement measures in Experiment 1.

	Question location (before vs. after)	Inference Contrast 1 (control vs. inference)	Inference Contrast 2 (inconsistent vs. inference)
Gaze duration	*b* = .03, *SE* = .02, *t* = 1.55, *p* > .1	*b* = .16, *SE* = .05, *t* = 3.38, *p* < .001*	*b* = .001, *SE* = .05, *t* = 0.01, *p* > .9
Go past time	*b* = .001, *SE* = .02, *t* = 0.06, *p* > .9	*b* = .12, *SE* = .05, *t* = 2.37, *p* < .05*	*b* = .002, *SE* = .05, *t* = 0.03, *p* > .9
Regressions out	*b* = .01, *SE* = .08, *z* = 0.10, *p* > .9	*b* = .10, *SE* = .20, *z* = 0.50, *p* > .6	*b* = .26, *SE* = .21, *z* = 1.24, *p* > .2
Regressions in	*b* = .10, *SE* = .08, *z* = 1.33, *p* > .1	*b* = .57, *SE* = .19, *z* = 2.95, *p* < .005*	*b* = .01, *SE* = .19, *z* = 0.05, *p* > .9
Total reading time	*b* = .04, *SE* = .02, *t* = 2.02, *p* < .02	*b* = .26, *SE* = .05, *t* = 5.80, *p* < .001*	*b* = .03, *SE* = .05, *t* = 0.59, *p* > .55
	Question × Inference Contrast 1	Question × Inference Contrast 2	Word reading efficiency
Gaze duration	*b* = .05, *SE* = .05, *t* = 1.14, *p* > .2	*b* = .03, *SE* = .05, *t* = 0.62, *p* > .5	*b* = .01, *SE* = .003, *t* = 2.55, *p* < .05*
Go past time	*b* = .004, *SE* = .05, *t* = 0.09, *p* > .9	*b* = .01, *SE* = .05, *t* = 0.11, *p* > .9	*b* = .02, *SE* = .004, *t* = 4.33, *p* < .001*
Regressions out	*b* = .15, *SE* = .19, *z* = 0.78, *p* > .4	*b* = .24, *SE* = .21, *z* = 1.13, *p* > .2	*b* = .03, *SE* = .01, *z* = 3.12, *p* < .005*
Regressions in	*b* = .33, *SE* = .19, *z* = 1.74, *p* = .08	*b* = .36, *SE* = .19, *z* = 1.97, *p* < .05	*b* = .02, *SE* = .01, *z* = 1.80, *p* = .07
Total reading time	*b* = .04, *SE* = .05, *t* = 0.84, *p* > .4	*b* = .09, *SE* = .05, *t* = 1.88, *p* = .06	*b* = .02, *SE* = .004, *t* = 4.66, *p* < .001*

*SE*: standard error.

* Two-tailed significance criterion (*t* or *z* ⩾ 2.29, *p* < .022), corresponding to a 5% error, with adjustment for multiple comparisons.

##### Question region

Table 9 shows gaze durations and total reading times, and Table 10 shows the results of the models. We saw large effects of question location in both measures, with much longer initial and total reading times on the question when it was presented before rather than after the passage. This is the opposite pattern to that observed in the antecedent region. Finally, there was an interaction between question location and inference condition, showing longer total reading times in the inference condition than the inconsistent condition when the question came before the text (*SE* = 0.05, *t* = 2.65, *p* < .01), but not when it came after it (*SE* = 0.06, *t* = 1.15, *p* > .2). In this region, more efficient word readers were faster in total reading times but not in gaze durations.

**Table 9.** Mean reading times and regression probabilities in the question region in Experiment 1.

	Question after text			Question before text		
	Control	Inference	Inconsistent	Control	Inference	Inconsistent
Gaze duration	948 (965)	1,052 (1,028)	905 (882)	2,227 (1,404)	2,108 (1,499)	1,953 (1,368)
Total reading time	1,849 (1,293)	1,885 (1,409)	1,898 (1,337)	3,104 (1,729)	3,122 (1,762)	2,787 (1,626)

Standard deviations in parentheses.

**Table 10.** Results for the question region examining the effect of inference and question location in gaze durations and total reading times in Experiment 1.

	Question location (before vs. after)	Inference Contrast 1 (control vs. inference)	Inference Contrast 2 (inconsistent vs. inference)
Gaze duration	*b* = .48, *SE* = .03, *t* = 15.59, *p* < .001*	*b* = .0003, *SE* = .08, *t* = 0.04, *p* > .9	*b* = .09, *SE* = .08, *t* = 1.20, *p* > .2
Total reading time	*b* = .25, *SE* = .02, *t* = 16.21, *p* < .001*	*b* = .004, *SE* = .04, *t* = 0.10, *p* > .9	*b* = .04, *SE* = .04, *t* = 1.00, *p* > .3
	Question × Inference Contrast 1	Question × Inference Contrast 2	Word reading efficiency
Gaze duration	*b* = .10, *SE* = .08, *t* = 1.27, *p* > .2	*b* = .05, *SE* = .08, *t* = 0.72, *p* > .4	*b* = .0003, *SE* = .01, *t* = 0.06, *p* > .9
Total reading time	*b* = .01, *SE* = .04, *t* = 0.35, *p* > .7	*b* = .10, *SE* = .04, *t* = 2.60, *p* < .01*	*b* = .01, *SE* = .004, *t* = 3.54, *p* < .001*

*SE*: standard error.

* Two-tailed significance criterion (*t* or *z* ⩾ 2.22, *p* < .026), corresponding to a 5% error, with adjustment for multiple comparisons.

### Discussion

Experiment 1 examined children’s spontaneous generation of local inferences during reading. Children exhibited longer first pass reading times in the inference than the control condition indicating that they made inferences online. Longer reading times on the anaphor in the inference condition can be taken to indicate increased processing difficulty, therefore supporting the hypothesis that children, like adults, make local inferences spontaneously and immediately while reading. In terms of comprehension monitoring, the critical comparison is between the inconsistent condition and the inference condition. During passage reading, there was no additional processing cost associated with encountering an inconsistent word over and above that caused by an inference, and indeed total reading times were shorter in the inconsistent than inference condition when the question was presented before the text. Overall, this suggests that children did make the inference as they read the passage but they either failed to detect the inconsistency or did not try to regulate their understanding by engaging in additional re-reading.

However, it is worth noting that requiring an inference to be made was not the only difference between the control and the other two conditions. In the control condition, the target word was a repetition of the antecedent (turtle-turtle). This raises the possibility that the shorter reading times in the control condition were due to the repeated word and the relative ease of processing associated with this, rather than difficulties associated with making an inference in the other two conditions. Although repeated words have also been used in previous studies examining inference making and anaphoric processing (Ehrlich et al., 1999; van der Schoot et al., 2008), with shorter reading times observed on them as compared with those requiring an inference, it is also the case that some experiments have reported a repeated word penalty. This occurs when a repeated word occurs where an anaphor was expected. This is judged to be unnatural and reading time slows, an effect that has been observed in children (Eilers et al., 2019). It is therefore unclear whether the differences in early reading times in the inference and control conditions reflect immediate inference making, a repeated word benefit, or a trade-off between a repeated word penalty and an inference.

The effects discussed so-far are those observed during first pass; that is, differences in reading times on the first encounter with the anaphor. While we cannot be sure whether longer gaze durations on the anaphor in the inference condition reflect online inference making, we can be confident that longer total reading times on the anaphor do reflect online inference making as we would not expect the effect of a repeated word to linger in these later measures. If this is the case and children generate this type of local inference only during later reading (i.e., the effect was only apparent once second and subsequent visits to the anaphor were incorporated into reading times), then this would show that inference making was not immediate but rather required time and re-visiting of the crucial parts of the passage for generation to occur. We will examine the possibility that the early effects were due to a simple word repetition effect, and later effects reflected inference generation, in Experiment 2.

For comprehension monitoring, the repeated word was not an issue as the key comparison (inference vs. inconsistent conditions) involved two non-repeated words. That there were no significant differences between these two conditions in either the antecedent or anaphor regions suggests that children were not doing anything more in terms of evaluation or regulation on encountering the anaphor beyond what they did when a consistent inference was required. Indeed, the only difference showed longer reading times in the inference than inconsistent condition; this provides further evidence that children did not detect the inconsistency. This is somewhat surprising given that the inconsistency manipulation was designed to be large. However, it may be the case that these processes are slow and not captured in analyses that target small and specific regions of interest. Scanpath analyses (von der Malsburg & Vasishth, 2011) might be more appropriate for investigating these temporally and spatially distributed effects. The design of the current experiment did not allow for this type of analysis.

We turn now to the effect of question location. Results from the antecedent (but not the anaphor) region showed significantly shorter reading times when the question was located before rather than after the text. Note that at this point in the passage, the information relevant to the questions has not yet occurred. This suggests that reading the question first leads to a strategy whereby children read over information more quickly that is not directly relevant to that question. However, this strategy appears to result in poorer comprehension of the texts, given that error rates were higher to questions that appeared before the text. Therefore, for local inferences at least, asking the question before reading had no benefit, and indeed appears to have cost global comprehension.

Interestingly, we saw the opposite pattern of effects on the question itself, with longer reading times on the question when it appeared before the passage rather than after it. There appears to be a trade-off whereby when the question appears first, children spent longer reading it and this then means they took less time to process the antecedent, perhaps because they were looking for the key information that they knew the question required. However, when children had not yet encountered the question, they spent longer reading the antecedent but then took less time reading the question when they came to it at the end of the passage. This might indicate they had built up an expectation of what might be asked. A reduction in reading times towards the end of a sentence or passage has been observed before (e.g., Schroeder, 2011) so these effects may simply reflect a more general reading pattern that is typically seen. That we saw no effect of question location in the anaphor region perhaps suggests that the difficulty of processing this region (which required an inference and/or the processing of inconsistent information in two of our three conditions) meant that the efficiency benefit of having already read the question was no longer apparent. In other words, reading the question first may lead to subsequent faster reading only when processing is relatively easy.

There was no interaction between question location and inference type during passage reading. We did see an interaction between question location and inference condition on the question itself, with longer total reading times in the inference than the inconsistent condition when the question came before the text, but not when it came after it. However, this is not of much theoretical interest as the question was simply shorter in the inconsistent condition. During passage reading, although having read the question first affected some aspects of children’s reading speed and response accuracy, there was no evidence that the placement of the question specifically led to a difference in spontaneous inference generation as we had predicted. Given that we did see evidence that children made the inferences, it is possible that this is because the inferences were local and therefore quite easy, and children did not need the potential benefit of reading the question first.

## Experiment 2

It is well-documented that local inferences are generally easier to make than global ones (Magliano et al., 1993; McKoon & Ratcliff, 1992), and it may be that while children make local inferences during reading, they do not spontaneously make global inferences as they read. If this is the case, question location might have a more pronounced effect as it would prompt children to make an inference which they otherwise would not do. We investigated this in Experiment 2. We also examined processing without the possible confound induced by having a repeated word in the control condition.

Our aims and predictions in Experiment 2 therefore differed somewhat to those in Experiment 1. In Experiment 2, rather than inferences being based on anaphor resolution within one or two sentences, children needed to integrate information across the paragraph (two target regions in particular) with their existing knowledge to successfully make the inference. Example stimuli for Experiment 2 are shown in Table 12. Adults only make global inferences online under certain (highly predictive) circumstances, or when there is a coherence break. We therefore expected that children would not make a global inference unless the question was presented first or there was an inconsistency in the passage. In terms of answering questions, we predicted that children would answer more questions correctly in the control than inference condition. As in Experiment 1, the question was different in the inconsistent condition and so was not directly comparable. We also predicted an interaction such that reading the question before the text would improve accuracy in the inference but not in the control condition.

In terms of the eye movement data, when the question was presented after the text, we predicted no differences in early or late eye movement measures across the inference conditions during passage reading: in the absence of a prompt to encourage children to make the inference, we expected that they would not do so spontaneously. In contrast, when the question appeared before the text, we predicted longer reading times in the second target region in the inference than control condition. We also predicted longer reading times on the anaphor in the inconsistent than the inference condition (*sunhat vs. umbrella* in Table 12) as we know that adults do make global inferences as they read when there is a coherence break (e.g., *dark grey clouds* followed by *sunhat* rather than *umbrella*). We further predicted that this inconsistency effect would be observed late in the eye movement record as we know that effects in children are often observed later than in adults (e.g., Joseph et al., 2008) and the more difficult global inferences might slow down comprehension monitoring and regulation processes.

Finally, we predicted that we might see effects of inference while reading the question itself as it might act as a prompt for children to make the inference. We expected to observe these effects differently across the two question locations: when the question was presented before the text, we expected to see longer total reading times on the question in the inference than the control condition due to additional re-reading. In contrast, when the question appeared after the text, we expected to see effects of the inference condition in first pass times as the children would have already read the text and might be prompted to make the inference on first encountering the question. Finally, in line with the results from Experiment 1, we predicted shorter reading times on the two clue regions and longer reading times on the question when the question appeared before the text, in line with Experiment 1.

### Method

#### Participants

Thirty-six children from Experiment 1 also took part in Experiment 2, alongside 17 additional children recruited from Years 5 and 6 classrooms, making 53 children in total (mean age = 10.7 years, *SD* = 1.4). Table 11 provides information on the children’s reading skills. All participants were monolingual native English speakers with no known reading difficulties. Each received a sticker and certificate to thank them for participating.

**Table 11.** Mean age and mean performance on standardised tests of reading and nonverbal IQ of children in Experiment 2.

	Year 4	Year 5	Year 6	Year 7	Year 8
Number (females)	12 (9)	18 (11)	8 (6)	11 (6)	5 (2)
Age	9.0 (0.3)	9.9 (0.4)	10.9 (0.3)	12.2 (0.3)	13.3 (0.3)
TOWRE words^a,b^	103 (3)	102 (14)	107 (16)	97 (10)	97 (12)
TOWRE nonwords^a,b^	100 (5)	108 (14)	117 (19)	101 (12)	104 (10)
YARC comprehension (primary)^a,c^	101 (11)	99 (11)	113 (6)	102 (9)	97 (8)
YARC comprehension (secondary)^a,c^	–	–	–	97 (9)	91 (9)
WASI matrices (t score)^d,e^	53 (14)	56 (20)	76 (NA)	57 (13)	50 (13)

Standard deviations are in parentheses.

a Standard score *M* = 100, *SD* = 15.

b TOWRE (Test of Word Reading Efficiency; Torgesen et al., 1999).

c YARC (York Assessment of Reading for Comprehension; Snowling et al., 2009).

d Standard score *M* = 50, *SD* = 10.

e WASI (Wechsler Abbreviated Scale of Intelligence, Wechsler, 1999).

#### Stimuli

As in Experiment 1, 24 short narrative passages were created (see Table 12) each between three and five sentences. The mean passage length was 205 characters (*SD* = 36). In all passages, the first sentence introduced a scenario. In the second or third sentence, a clue region appeared (*dark grey clouds* in Table 12). This region was identical across the three conditions. The following sentence contained the critical region (*umbrella* or *sunhat* in Table 12). This region was identical across the control and inference conditions but different in the inconsistent condition. Word length and frequency were controlled across conditions (*p*s > .4). The inference and control conditions differed only in the first or second sentence preceding the clue region in which explicit information needed to answer the question was given in the control (*It was going to rain* in Table 12) but not the inference condition.

**Table 12.** Example stimuli for Experiment 2. In each of the three versions of the passage, the first clue region is in italics and the second clue region is underlined.

	Story	Question
*Control condition*	Mr. Jones was on his way to a meeting. It was going to rain. He looked up at the *dark grey clouds* in the sky. He let out a big sigh and he put his umbrella up sadly.	Why did Mr. Jones put his umbrella up? a. it was going to rain b. he let out a big sigh c. protect him from the sun
*Inference condition*	Mr. Jones was on his way to a meeting and it was almost 5 o’clock. He looked up at the *dark grey clouds* in the sky. He let out a big sigh and put his umbrella up sadly.	Why did Mr. Jones put his umbrella up? a. it was going to rain b. he let out a big sigh c. protect him from the sun
*Inconsistent condition*	Mr. Jones was on his way to a meeting and it was almost 5 o’clock. He looked up at the *dark grey clouds* in the sky. He let out a big sigh and put his sunhat on sadly.	Where was Mr. Jones going? a. was almost 5 o’clock b. the library c. a meeting

A question was associated with each passage. As in Experiment 1, the questions for the control and inference conditions were identical but the question for the inconsistent condition was different. For inconsistent passages, the question related to information encountered before the inconsistency. For the control passage, the correct response to the question was explicitly stated in the text. Arguably, children still needed to make an inference in that they needed to connect this information with the target word (e.g., rain-umbrella) but the inference demand was more substantial in the inference condition: the correct response required children to make a global inference using the clue regions and general knowledge (i.e., if the clouds were grey and Mr. Jones put his umbrella up then it was likely that it was going to rain). The location of the question was experimentally manipulated such that the question appeared before or after the passage. As in Experiment 1, all passages and stimuli were pre-screened with ten 7- to 11-year-olds to ensure that passages were age-appropriate and that the majority of questions could be answered correctly. Small changes were made to the passages following this. Final stimuli for Experiment 2 are available at https://osf.io/ngjra/.

#### Apparatus and procedure

The apparatus and procedure were the same as in Experiment 1. For those children who had also completed Experiment 1, the order in which the two experiments were completed was counterbalanced across the 2 days of testing.

### Results

Data were analysed in the same way as in Experiment 1.

#### Answers to the comprehension questions

Table 13 shows response accuracy to comprehension questions presented before or after the passages. A model with inference type and question location as fixed factors and participant and item as random factors revealed no main effects or interactions (*z*s < 1).

**Table 13.** Proportion of incorrect responses to comprehension questions in Experiment 2.

	Control	Inconsistent	Inference
After	0.07 (0.26)	0.10 (0.30)	0.10 (0.30)
Before	0.08 (0.28)	0.09 (0.28)	0.09 (0.29)

Standard deviations in parentheses.

#### Eye movements

As in Experiment 1, we ran models with two fixed factors: Inference (control, inference, or inconsistent) and Question Location (before or after text) for each eye movement measure of interest in all three regions of interest. Word reading efficiency was added to all models as a covariate.

##### First clue region

Table 14 shows the mean reading times and proportion of regressions in the first clue region, and Table 15 shows the results of the models. There was an effect of question location with longer reading times for gaze durations and total reading times when the question was presented after the text. Gaze durations were also longer in the inference than the control condition, but this effect was not observed in any other measures. There were no other effects of inference and no interactions. Children with more efficient word reading showed faster reading times for all measures; they also showed slightly fewer regressions out of the region.

**Table 14.** Reading times and regression probabilities for first target region (Clue 1) in Experiment 2.

	Question after text			Question before text		
	Control	Inference	Inconsistent	Control	Inference	Inconsistent
Gaze duration	546 (466)	488 (456)	508 (447)	398 (259)	457 (371)	401 (314)
Go past time	743 (595)	735 (701)	673 (503)	710 (781)	787 (817)	735 (641)
FP regressions out	0.29 (0.46)	0.25 (0.43)	0.25 (0.43)	0.29 (0.45)	0.28 (0.45)	0.33 (0.47)
Regressions in	0.24 (0.43)	0.21 (0.40)	0.27 (0.44)	0.20 (0.40)	0.18 (0.39)	0.24 (0.43)
Total reading time	710 (628)	719 (578)	681 (551)	605 (412)	665 (591)	637 (511)

Standard deviations in parentheses.

**Table 15.** Results for first clue region examining the effect of inference, and question location on all eye movement measures in Experiment 2.

	Question location (before vs. after)	Inference Contrast 1 (control vs. inference)	Inference contrast 2 (inconsistent vs. inference)
Gaze duration	*b* = .07, *SE* = .02, *t* = 3.54, *p* < .001*	*b* = .02, *SE* = .04, *t* = 3.38, *p* < .001*	*b* = .02, *SE* = .05, *t* = 0.53, *p* > .5
Go past time	*b* = .01, *SE* = .02, *t* = 0.74, *p* > .4	*b* = .02, *SE* = .05, *t* = 0.50, *p* > .6	*b* = .03, *SE* = .05, *t* = 0.62, *p* > .5
Regressions out	*b* = .01, *SE* = .08, *z* = 0.10, *p* > .9	*b* = .10, *SE* = .20, *z* = 0.50, *p* > .6	*b* = .26, *SE* = .21, *z* = 1.24, *p* > .2
Regressions in	*b* = .09, *SE* = .08, *z* = 1.20, *p* > .2	*b* = .15, *SE* = .19, *z* = 0.78, *p* > .4	*b* = .37, *SE* = .19, *z* = 1.98, *p* < .05
Total reading time	*b* = .04, *SE* = .02, *t* = 2.66, *p* < .01*	*b* = .03, *SE* = .04, *t* = 0.77, *p* > .4	*b* = .03, *SE* = .04, *t* = 0.79, *p* > .4
	Question × Inference Contrast 1	Question × Inference Contrast 2	Word reading efficiency
Gaze duration	*b* = .10, *SE* = .04, *t* = 2.19, *p* < .05	*b* = .07, *SE* = .05, *t* = 1.43, *p* > .1	*b* = .02, *SE* = .002, *t* = 6.90, *p* < .001*
Go past time	*b* = .09, *SE* = .05, *t* = 2.04, *p* < .05	*b* = .03, *SE* = .05, *t* = 0.67, *p* > .5	*b* = .02, *SE* = .002, *t* = 8.00, *p* < .001*
Regressions out	*b* = .15, *SE* = .19, *z* = 0.78, *p* > .4	*b* = .24, *SE* = .21, *z* = 1.13, *p* > .2	*b* = .03, *SE* = .01, *z* = 3.12, *p* < .005*
Regressions in	*b* = .10, *SE* = .19, *z* = 0.52, *p* > .6	*b* = .03, *SE* = .19, *z* = 0.19, *p* > .8	*b* = .01, *SE* = .01, *z* = 1.72, *p* = .09
Total reading time	*b* = .01, *SE* = .04, *t* = 0.16, *p* > .8	*b* = .01, *SE* = .04, *t* = 0.26, *p* > .8	*b* = .02, *SE* = .002, *t* = 8.78, *p* < .001*

*SE*: standard error.

* Two-tailed significance criterion (*t* or *z* ⩾ 2.29, *p* < .022), corresponding to a 5% error, with adjustment for multiple comparisons.

##### Second clue region

Table 16 shows the mean reading times and regression probabilities in the second clue region, and Table 17 shows the results of the models. There were longer gaze durations, go past times, and total times when the question was presented after the text.

**Table 16.** Reading times and regression probabilities for the second target region (Clue 2).

	Question after text			Question before text		
	Control	Inference	Inconsistent	Control	Inference	Inconsistent
Gaze duration	505 (443)	470 (438)	541 (574)	418 (301)	465 (495)	390 (347)
Go past time	799 (669)	734 (676)	941 (992)	581 (478)	664 (635)	712 (767)
FP regressions out	0.30 (0.46)	0.28 (0.45)	0.33 (0.47)	0.23 (0.42)	0.29 (0.46)	0.28 (0.45)
Regressions in	0.30 (0.46)	0.31 (0.46)	0.33 (0.47)	0.36 (0.48)	0.19 (0.39)	0.33 (0.47)
Total reading time	907 (954)	816 (823)	931 (791)	586 (432)	664 (674)	765 (743)

Standard deviations in parentheses.

**Table 17.** Results for second clue region examining the effect of inference and question location in children on all eye movement measures in Experiment 2.

	Question location (before vs. after)	Inference Contrast 1 (control vs. inference)	Inference Contrast 2 (inconsistent vs. inference)
Gaze duration	*b* = .06, *SE* = .02, *t* = 2.98, *p* < .005*	*b* = .02, *SE* = .05, *t* = 0.32, *p* > .7	*b* = .03, *SE* = .05, *t* = 0.69, *p* > .5
Go past time	*b* = .09, *SE* = .02, *t* = 4.30, *p* < .001*	*b* = .01, *SE* = .05, *t* = 0.41, *p* > .8	*b* = .09, *SE* = .05, *t* = 1.66, *p* = .1
Regressions out	*b* = .06, *SE* = .08, *z* = 0.78, *p* > .4	*b* = .09, *SE* = .20, *z* = 0.45, *p* > .6	*b* = .18, *SE* = .20, *z* = 0.88, *p* > .3
Regressions in	*b* = .05, *SE* = .07, *z* = 0.64, *p* > .5	*b* = .45, *SE* = .18, *z* = 2.47, *p* < .05*	*b* = .49, *SE* = .18, *z* = 2.69, *p* < .01*
Total reading time	*b* = .11, *SE* = .02, *t* = 5.82, *p* < .001*	*b* = .05, *SE* = .05, *t* = 0.97, *p* > .3	*b* = .18, *SE* = .05, *t* = 3.76, *p* < .001*
	Question × Inference Contrast 1	Question × Inference Contrast 2	Word reading efficiency
Gaze duration	*b* = .02, *SE* = .05, *t* = 0.41, *p* > .6	*b* = .08, *SE* = .05, *t* = 1.65, *p* = .1	*b* = .02, *SE* = .003, *t* = 6.87, *p* < .001*
Go past time	*b* = .01, *SE* = .05, *t* = 0.21, *p* > .8	*b* = .06, *SE* = .05, *t* = 1.09, *p* > .2	*b* = .03, *SE* = .003, *t* = 10.33, *p* < .001*
Regressions out	*b* = .28, *SE* = .20, *z* = 1.38, *p* > .1	*b* = .14, *SE* = .20, *z* = 0.72, *p* > .4	*b* = .01, *SE* = .01, *z* = 1.63, *p* > .1
Regressions in	*b* = .52, *SE* = .18, *z* = 2.83, *p* < .005*	*b* = .35, *SE* = .18, *z* = 1.89, *p* = .06	*b* = .03, *SE* = .01, *z* = 3.26, *p* < .005*
Total reading time	*b* = .01, *SE* = .05, *t* = 0.15, *p* > .8	*b* = .04, *SE* = .05, *t* = 0.77, *p* > .4	*b* = .03, *SE* = .002, *t* = 10.54, *p* < .001*

*SE*: standard error.

* Two-tailed significance criterion (*t* or *z* ⩾ 2.29, *p* < .022), corresponding to a 5% error, with adjustment for multiple comparisons.

Unexpectedly, we also saw evidence in this region that children were doing something different in the inference condition: they made more regressions into this region in the control than the inference condition, and in the inconsistent than the inference condition. That is, they made substantially fewer regressions in the inference than the other two conditions. They also showed longer total reading times in the inconsistent than inference condition. There was also an interaction between inference condition and question location showing fewer regressions into the region in the inference than the control condition when the question was presented before (*b* = .48, *SE* = .14, *z* = 3.34) but not after (*b* = .01, *SE* = .06, *z* = 0.08) the passage. Finally, children with better word reading showed faster reading times (gaze durations, go past times, and total reading times) and made fewer regressions into this region.

##### Question region

Table 18 shows the mean reading times and proportion of regressions in the question region, and Table 19 shows the results of the models. There were large effects of question location (with longer reading times when the question came before the passage) in gaze durations and total reading times but no effects of inference and no interactions. Children with more proficient reading (as measured by the standardised test) also showed shorter gaze durations and total reading times.

**Table 18.** Reading times and regression probabilities for the question region.

	Question after text			Question before text		
	Control	Inconsistent	Inference	Control	Inconsistent	Inference
Gaze duration	1,029 (851)	1,034 (974)	1,016 (974)	1,830 (1,045)	1,990 (1,220)	1,737 (1,139)
Total reading time	1,566 (961)	1,797 (1,338)	1,786 (1,075)	2,464 (1,294)	2,631 (1,499)	2,379 (1,319)

Standard deviations in parentheses.

**Table 19.** Results for question region examining the effect of inference and question location in children on all eye movement measures in Experiment 2.

	Question location (before vs. after)	Inference Contrast 1 (control vs. inference)	Inference Contrast 2 (inconsistent vs. inference)
Gaze duration	*b* = .40, *SE* = .03, *t* = 14.94, *p* < .001*	*b* = .12, *SE* = .07, *t* = 1.82, *p* = .07	*b* = .12, *SE* = .07, *t* = 1.84, *p* = .07
Total reading time	*b* = .20, *SE* = .01, *t* = 14.06, *p* < .001*	*b* = .04, *SE* = .03, *t* = 1.15, *p* > .2	*b* = .03, *SE* = .03, *t* = 0.77, *p* > .4
	Question × Inference Contrast 1	Question × Inference Contrast 2	Word reading efficiency
Gaze duration	*b* = .005, *SE* = .06, *t* = 0.07, *p* > .9	*b* = .05, *SE* = .07, *t* = 0.65, *p* > .4	*b* = .01, *SE* = .003, *t* = 3.30, *p* < .005*
Total reading time	*b* = .06, *SE* = .03, *t* = 1.69, *p* = .09	*b* = .04, *SE* = .03, *t* = 1.15, *p* > .2	*b* = .02, *SE* = .002, *t* = 10.34, *p* < .001*

*SE*: standard error.

* Two-tailed significance criterion (*t* ⩾ 2.22, *p* < .026), corresponding to a 5% error, with adjustment for multiple comparisons.

### Discussion

Experiment 2 set out to answer two main questions: first, do we see evidence of online inference making when there is no repeated word and the inferences are global rather than local, and second, does question location affect reading behaviour in general and help readers to make inferences specifically?

The results from Experiment 2 show that question location makes a difference to how children read a passage, with longer reading times on the question itself when it appears before the text, and longer reading times on target regions within the passage when the question is presented after the text. These findings echo those from Experiment 1, although note that pattern was seen in both target regions in Experiment 2 but only in the first target region in Experiment 1. As in Experiment 1, although reading patterns changed when the question was presented before the text, this was not associated with any benefit for global comprehension: spending longer reading the question didn’t make answers more likely to be accurate, and by implication global understanding of the text stronger. Unlike Experiment 1 where performance on comprehension questions was worse when the question was presented before the text, accuracy was similar in Experiment 2 regardless of question location (or inference condition).

In relation to inference making, the pattern of results is complex but overall there is no clear and compelling evidence of spontaneous inference making during reading. Although children spent longer reading the first clue region in the inference than control condition during first pass, suggesting spontaneous early inference generation, we are wary of this effect as it is not observed in any other measures or regions. At best, we suggest it represents a fleeting effect, perhaps reflecting partial inference making. Alternatively, it is possible that it does not reflect inference making at all and can be dismissed as an anomaly in the data. We know from many previous studies that higher level processes such as inference generation are usually observed over a number of eye movement measures and regions of interest. It therefore seems unlikely that in our experiment, there are effects of inference that are immediate and short-lived, especially given the much longer-lasting effects observed in Experiment 1. It is also hard to see how taking longer to read the first clue word region in the inference condition might constitute inference generation. If anything, we might expect longer gaze durations in the control condition as children linked the clue region (e.g., *dark grey clouds*) with the previously encountered explicit information (e.g., *rain*). In the inference condition, spending longer reading the clue would indicate rapid and spontaneous predictive inference generation (without a constraining context); this has not been observed before in adult readers (Cook et al., 2001) much less in children.

Although there was no convincing evidence of an inference effect in the direction we predicted, we did see increased processing effort in both the control and inconsistent conditions compared with the inference condition. This suggests that children were integrating the target word into their ongoing situation model to a greater degree in these two conditions than in the inference condition. It follows from this that they were not making an inference unless information was explicit (i.e., the control condition stated that it was going to rain), or there was inconsistent information (i.e., that Mr. Jones put on his sunhat). This interpretation is in line with the proposition that global inferences are made only when there is a coherence break (McKoon & Ratcliff, 1992).

Although there were no other main effects of inference condition, there was an interesting interaction between question location and inference condition showing that if the question had been encountered previously, children made more regressions into the second clue region in the control condition relative to the inference condition. However, there was no difference across passage type if they had not yet encountered the question. This provides further evidence that children did not make inferences spontaneously; rather, encountering the question helped them to link the explicit information (rain) with the target word (umbrella). That is, in the easiest condition, children were linking information across the passage (i.e., making a local inference), but this was not the case in the more difficult conditions (when the question appeared after the text or when a global inference was required). It should be noted that although we categorised inferences as global in Experiment 2, the distance between the clue words was smaller than in a number of previous studies (e.g., Long & Chong, 2001) and so it could be argued that the information needed to make the inference may still be available to working memory. Nevertheless, it is certainly the case that more work was needed to generate these inferences than those in Experiment 1.

In terms of comprehension monitoring, there was evidence that children’s reading was disrupted on encountering *sunhat* rather than *umbrella*, suggesting that this inconsistency prompted them to make the inference. Specifically, children made more regressions into *sunhat* and then spent longer reading it in total compared with the inference condition. This suggests that although their evaluation and regulation were not immediate, increased difficulty in processing was present, in contrast to the inference-control comparison. Note however some caution is warranted. Although the differences between the inference and inconsistent conditions were statistically significant, the reading times and regression probabilities were broadly similar in the control and inconsistent conditions, but reading times were shorter in the inference condition, and fewer regressions were made. Rather than an inconsistency effect, it might be that the inference condition was easier than the other two, presumably because children did not detect that an inference was required.

As there were more regressions into the second clue region in the inconsistent than the inference condition when the question came before the text, one could make a case that reading the question first helped the children to make a partial inference. On this view, encountering *sunhat* as compared with *umbrella* after reading about *dark grey clouds* resulted in disruption to processing, but their situation model was not sufficiently specified to drive re-analysis. Once again, however, we feel that caution is needed. A more conservative interpretation is that question location did not make a difference to comprehension monitoring and children detected the inconsistency to the same degree in both question location conditions. We conclude therefore that our results provide no strong evidence that children aged 8 to 13 spontaneously generate global inferences online unless there is a coherence break, even when they were prompted to do so by the question appearing before the text.

## General discussion

Generating inferences and monitoring comprehension are two critical components of reading comprehension. While there have been many studies of children’s inference making and comprehension monitoring using offline methods, we know relatively little about when during (or after) reading children make different types of inferences and detect inconsistencies, and whether prompts such as questions affect the time course in which they are made. We conducted two experiments with 8- to 13-year-old children to examine (1) whether they make local and global inferences online as they read, (2) whether they evaluate and regulate their ongoing comprehension as they read, and (3) whether presenting a question that taps an inference before reading a text prompts children to make an inference during reading that they otherwise would not. We address each of these questions in turn.

### Online inference generation

Our results suggest that children spontaneously make local inferences as they read, but not global inferences. In Experiment 1, we observed both early (gaze durations) and later (total reading times reading and regressions) effects of local inference generation on the anaphor that was a semantic category (*reptile*) for its antecedent (*turtle*) rather than a repetition of it. We will first consider the early effects as these could be interpreted in two ways: as early indicators of inference generation or as word repetition effects. There are three reasons to suspect the latter. First, in a previous experiment with a similar design but no repeated word (Joseph et al., 2013), no effect was observed during first pass: the earliest measure in which effects were seen was in regressions out of the anaphor. Second, higher level effects (i.e., effects beyond the lexical level) tend to be seen later in the eye movement record of children compared with adults. This is seen both in terms of eye movement measures themselves (e.g., effects seen in gaze duration for adults are not seen until total time in children) and where in the text they occur: in children, effects are often one or two words downstream of the target region (e.g., Joseph et al., 2008). Third, the apparent effect may have been due to the repeated word (*turtle*) rather than the generation of a local inference. A clear next step from this evaluation would be to analyse the post-anaphor region in both experiments to see if effects of the inference manipulation were evident here. However, this is not possible in our dataset as the stimuli were not created with this in mind—too often there are only one or two words between the second target region and the end of the sentence. We urge other researchers to consider post-target regions in future studies with children.

Moving now to the later effects observed, the first reliable sign of children making a local inference was observed in total reading times on the anaphor, and regressions into the anaphor. At this point, children spent longer reading, re-visiting, and re-reading the anaphor in the inference than the control condition. It is important to note that total reading times encompass both initial and later visits to the word. If the longer gaze durations observed in the inference condition when first encountering the anaphor was consequence of a word repetition effect as discussed above, causing children in some cases to go back and reinspect earlier portions of the text (indexed by longer go past times), then we need to look to later measures to find evidence of inference generation. Examining differences between first pass (gaze durations) and total reading times, children did indeed revisit the anaphor more often and for longer (112 ms) in the inference than control condition during second pass. A clearer way to examine this late reading time effect would have been to examine second pass reading times rather than making assumptions based on the difference between total reading times and gaze durations. However, this was not possible in the current study due to a large proportion of missing data. Nevertheless, the large difference in total reading times on the anaphor, together with the increase in regressions, in the inference condition provides compelling evidence that children at least attempted to make a local inference at this point. Indeed, given the high performance on the comprehension questions, it is likely that they were generally successful in doing so.

In sum, we know that skilled adult readers make local inferences online as they read and that these can be observed in early reading time measures, namely gaze durations (e.g., Duffy & Rayner, 1990). Our results suggest that children aged 8 to 13 years also make local inferences online but do so with a delayed time course. As has been observed across a number of studies, post-lexical processing is generally observed later in the eye movement record for children as compared with adults (Joseph et al., 2008; Joseph & Liversedge, 2013). Our results therefore add to this growing literature that children process written language in a similar way to adults but simply do so more slowly. We know also from previous studies (Blythe et al., 2006; Joseph et al., 2009; Kirkby et al., 2008; McConkie et al., 1991) that this is not due to oculomotor control or slower visual processing (i.e., extracting the visual information from words needed to embark on linguistic processing) but rather lexical and post-lexical processing is more laborious for children who have less experience and expertise in reading (Reichle et al., 2013). Although we were not able to do so in the current studies due to substantial variability in length across items, we do encourage other researchers to examine eye movement behaviour on the post-target region in passages that require the generation of inferences as it may be that effects are observed at this point.

Moving now to global inferences, there was no compelling evidence for online generation of this type of inference. Although we observed longer gaze durations in the inference than control condition in the first clue region, it is unlikely that readers would generate a predictive inference at this early point in the text, especially given the likely delayed effects observed in Experiment 1. In addition, this pattern was not observed in any other measure or region, and it is important that an isolated effect is not over-interpreted. In the second clue region, reading times were substantially shorter in the inference than the control and inconsistent conditions; this faster reading time suggests that children do not make global inferences as they read. The children only made links between previously encountered information and currently processed text when inference demands were low (linking *rain* with *umbrella* in the control condition) or when there was a coherence break (*sunhat* rather than *umbrella*). This is consistent with the view that children, like adults (Calvo et al., 2001; O’Brien et al., 1988), do not engage in non-essential inference generation under normal conditions, as stated by the minimalist theory of inference generation (McKoon & Ratcliff, 1992).

Although we did not expect to see spontaneous inference making in children without prompts or coherence breaks, in line with what we know from the adult literature (e.g., O’Brien et al., 1988), we did expect the presence of a question before reading to prompt children to process the passage differently, thereby making it more likely that a global inference would be made, even in the absence of an inconsistency. This was not seen: reading the question first had no influence on global inference generation. Clearly children can make global inferences as this is seen in many offline experiments that ask children to answer questions that tap global inference (e.g., Cain & Oakhill, 1999). It might be that a strong cue is needed for a global inference to be made—like asking a question that prompts re-analysis and re-evaluation.

An alternative explanation is that our design and sample size did not allow us to capture what may be a small effect that varies greatly across individuals. Previous studies have shown that individual differences in comprehension skill are predictive of high-level language processing, including inference generation (Joseph et al., 2015; van der Schoot et al., 2008) and comprehension monitoring (Connor et al., 2015; van der Schoot et al., 2009, 2012). As our sample was relatively small but varied in age and in reading skill, it may be the case that older and more skilled readers were generating inferences as they read but younger and less skilled readers were not. Indeed, the pattern of reading times in both clue regions when the question came first are suggestive of increased processing difficulty in the inference as compared with the control condition. Across experiments, reading time measures, and regions of interest, we very consistently saw that children who scored highly in our TOWRE were faster readers and made fewer regressions. Given that we were able to capture this variability in our models, it is likely that these individual differences interacted with our fixed effects. However, it was not possible to examine this possibility in the current data set due to its relatively small sample and complex design. We hope that by making our data freely available, it will be possible to combine data with other researchers and that future studies will be able to investigate these important questions.

### Comprehension monitoring

Our design allowed us to examine comprehension monitoring in conjunction with inference making: inconsistency in the passage should only be detected if the children had made the appropriate inference. For example, a man wearing a sunhat is not in itself strange, but if it had been inferred that is was likely to rain (based on reading that there were dark clouds in the sky) then wearing a sunhat would be inconsistent. Furthermore, we reasoned that the explicit nature of the inconsistency would mean that only a partial inference was needed for it to be detected. On this logic, it would be possible to see inconsistency, but not inference effects. This, we argue, would be evidence that children were making partial or underspecified inferences.

There was no evidence of sensitivity to inconsistency in Experiment 1. In Experiment 2, however, there was evidence of comprehension monitoring. Longer total reading times and more regressions into the second clue region in the inconsistent than the inference condition indicate that there was an additional processing cost associated with integrating an inconsistent word into the ongoing situation model. This pattern of results fits with the idea that inconsistency effects emerge in the absence of a fully formed inference. This may have been the case and given what we know about comprehension monitoring being harder when inconsistent pieces of information are nonadjacent in a text (Yuill et al., 1989) and it requiring the integration of propositions to construct a coherent representation of a text (Cain et al., 2004), it is clear why detecting inconsistencies would be particularly challenging in Experiment 2. However, we must also acknowledge that numerically reading times and regression frequencies were equivalent across the control and inconsistent conditions, suggesting that they were of similar difficulty, although we did not compare these two conditions directly. It is therefore possible that the inconsistency was not detected, rendering this condition equivalent to the control condition, while the inference condition was easier than both the other two. Theoretically, it is difficult to explain this interpretation, but it remains a possibility.

Across our two experiments then, we see a somewhat mixed picture of spontaneous comprehension monitoring. When an inference is local and therefore easier to draw, encountering a word that is inconsistent with the text premise does not result in visible processing costs. This suggests that readers did not detect the inconsistence, perhaps because the text was easy to understand, local inferences were easy to make, and they therefore engaged in shallow good-enough processing (in which language processing is partial and semantic representations are often incomplete; Ferreira et al., 2002). Alternatively, they may have detected the inconsistency, but this was not captured in our analyses due to their focus on small, specific regions of the sentence. However, when the inference is global, although we see no clear evidence that an inference is made when the text is consistent, it seems that the break in coherence introduced by the inconsistency causes a processing cost which is observed as children read the text. Consistent with the adult literature (e.g., Cook & Myers, 2004), the current study provides some tentative evidence that children monitor their comprehension online when comprehension is challenging and requires close attention.

### Question location

Our final research question asked whether reading the question before the text resulted in more spontaneous inference generation. The answer to this question is no. In Experiment 1, our findings suggest that children made the local inference anyway—this meant there was no opportunity for the pre-reading question to prompt inference generation. Experiment 2 provided tentative evidence that question location may influence the time course of inference generation. In the first clue region, there was a tendency for longer reading times in the inference condition than the control condition, if the question came before (but not after) the passage; but, the opposite pattern was seen in the second clue region (longer reading times in the control than the inference condition) in the pre-reading question condition. Potentially, question location might influence inference generation, but in our data at least, the effect is subtle, fleeting, and hard to interpret. Future studies should seek to examine this possibility in more detail.

Considering the main effect of question location, we saw large and consistent effects across both experiments. If the question was presented before the text, children took more time to read it than when the same question appeared after the text. In contrast, the opposite pattern was seen during passage reading: if the question was presented first, children spent much less time reading the two target regions than if they had not yet encountered the question. This shows that children are sensitive to text structure and adapt their reading behaviour accordingly. This is likely to be because reading the question first induces a more purposeful reading strategy, such that once read, the passage itself is more predictable and easier to process. Although a similar pattern has been observed in adult readers (Kaakinen et al., 2015), it has not been reported previously in children (who tend to be less sensitive to questioning before or during reading; Kaakinen et al., 2015; Schumacher et al., 1983; van den Broek et al., 2001). Interestingly, however, spending longer reading the question (when it came first) was not associated with better performance on the comprehension question (also observed in Kaakinen et al., 2015): children answered fewer questions correctly in the question first condition in Experiment 1, and there was no difference in Experiment 2. Overall then we conclude that reading the question first does not help (and may hinder) global comprehension.

One explanation for this lack of benefit may be that reading the question first places a burden on working memory and this then affects subsequent comprehension. There is now much evidence that working memory and reading comprehension are associated and interactive (e.g., Cain et al., 2004; Nation et al., 1999; van Dyke et al., 2014). Relatedly, van den Broek and colleagues (2001) argued that adults and more proficient readers are able to direct their attention strategically and hence use questions to make connections with relevant parts of the text, thereby increasing the strength of their representations of the text meaning (relevant to the question). For younger readers, with limitations in their working memory resources, having the question in mind may interfere with ongoing processing of text and therefore impede comprehension. Based on our data, we would not wish to recommend that educators present questions before reading as a strategy to promote reading comprehension.

### Open science and pre-registration

Before closing, we return to the discussion of open science and pre-registration, initiated in the “Introduction” section. With numerous eye movement measures and multiple regions of interest being available in experiments such as ours, we chose to focus on main effects and interactions which could be considered in the context of previous work. We also took a conservative approach by correcting for multiple comparisons, as recommended by von der Malsburg and Angele (2017). We had hoped to investigate whether and how variation in eye movement behaviour is associated with individual differences in reading comprehension, as estimated by a standardised test. However, this induced too much analytic flexibility given we had not pre-registered our hypotheses or analysis plan. Our full dataset is available (https://osf.io/ngjra/) and may offer a resource for secondary analysis and generating exploratory observations that can then be tested out in appropriately powered and pre-registered experiments.

## Conclusion

Two experiments examined online inference making and comprehension monitoring in children. Our results show clear evidence that children make local inferences online, but not global inferences, and that location of the question does not affect these aspects of reading behaviour. While question location had a substantial effect on passage reading as revealed by the eye movement record, it had no effect on comprehension performance in one experiment and a detrimental effect in the other. While children did not draw global inferences while they are reading, they were made when answering questions. Like adults in previous studies, children appear to prioritise efficiency over completeness and make inferences only if and when necessary thereby freeing up resources for fluent, good-enough processing.
